# A rare case of *Listeria monocytogenes* bacteremia complicated by severe hyperbilirubinemia and liver failure

**DOI:** 10.1002/kjm2.12920

**Published:** 2024-12-10

**Authors:** Yuan‐Ai Tseng, Sheau‐Fang Yang, Tyng‐Yuan Jang

**Affiliations:** ^1^ School of Post‐Baccalaureate Medicine, Kaohsiung Medical University Kaohsiung Taiwan; ^2^ Department of Pathology Kaohsiung Medical University Hospital Kaohsiung Taiwan; ^3^ Department of Pathology School of Medicine, College of Medicine, Kaohsiung Medical University Kaohsiung Taiwan; ^4^ Hepatobiliary Division, Department of Internal Medicine Kaohsiung Medical University Hospital Kaohsiung Taiwan

*Listeria monocytogenes* primarily causes central nervous system (CNS) infections, but is rarely linked to the liver. Infections are often foodborne, commonly associated with contaminated foods, such as unpasteurized dairy, deli meat, and raw vegetables, and can lead to severe bacteremia and sepsis, particularly in immunocompromised patients, pregnant women, and the elderly. Here, we present a case of *L. monocytogenes* bacteremia complicated by unusual hyperbilirubinemia that subsequently progressed to liver failure.

A 55‐year‐old immunocompromised man with a history of hypertension presented with fever of 39.7°C 1 day before admission. The patient denied any history of hepatic disease and had no known alcohol consumption, smoking, and drug use habits. Furthermore, he recalled recent consumption of *zongzi* (a traditional meat‐stuffed rice dumpling), a potential foodborne source of *L. monocytogenes*. Physical examination revealed tenderness in the right upper quadrant with a positive Murphy's sign. Initial laboratory results were significant for leukocytosis (white blood cell count; 20,940/μL), elevated C‐reactive protein (262.23 mg/L), and elevated liver enzyme (Aspartate aminoTransferase (AST): 257 unit/L; Alanine aminoTransferase (ALT): 146 unit/L). Abdominal computed tomography demonstrated cholelithiasis and gallbladder wall edema; however, cirrhosis was not diagnosed. Blood cultures tested positive for *L. monocytogenes* and empirical antibiotic therapy with ampicillin and gentamicin was initiated. Because of the absence of CNS involvement, lumbar puncture was not performed. Magnetic resonance cholangiopancreatography revealed cholelithiasis without biliary dilation, liver cirrhosis, or splenomegaly, with no evidence of liver abscess (Figure 1A). The patient's fever resolved rapidly with antibiotic treatment and he reported no abdominal pain. However, repeat laboratory tests showed worsened liver enzymes (AST: 731 U/L; ALT: 112 U/L), hyperbilirubinemia (total bilirubin: 11.49 mg/dL; direct bilirubin: 9.3 mg/dL), hypoalbuminemia (2.38 g/dL), and prolonged prothrombin time (international normalized ratio (INR): 1.32). Further workup showed no viral hepatitis, autoimmune hepatitis, hemochromatosis, or Wilson's disease and no evidence of hepatitis A virus, hepatitis B virus, hepatitis C virus, or cytomegalovirus infections. Despite treatment, the patient developed worsening jaundice and continuously increasing serum bilirubin levels (total bilirubin: 13.4 mg/dL; direct bilirubin: 10.4 mg/dL), along with hypoalbuminemia (2.28 g/dL) and prolonged prothrombin time (INR: 1.42). Notably, the patient did not report any subjective discomfort. To investigate the cause of the persistent bilirubin elevation, a liver biopsy was performed. Pathological examination revealed acute hepatitis with ballooning degeneration of hepatocytes and marked bridging fibrosis with occasional cirrhotic nodules. (Figure 1B–D) No evidence of bleeding was observed in the liver biopsy. The patient's mortality was related to complications of acute liver failure.

**FIGURE 1:**
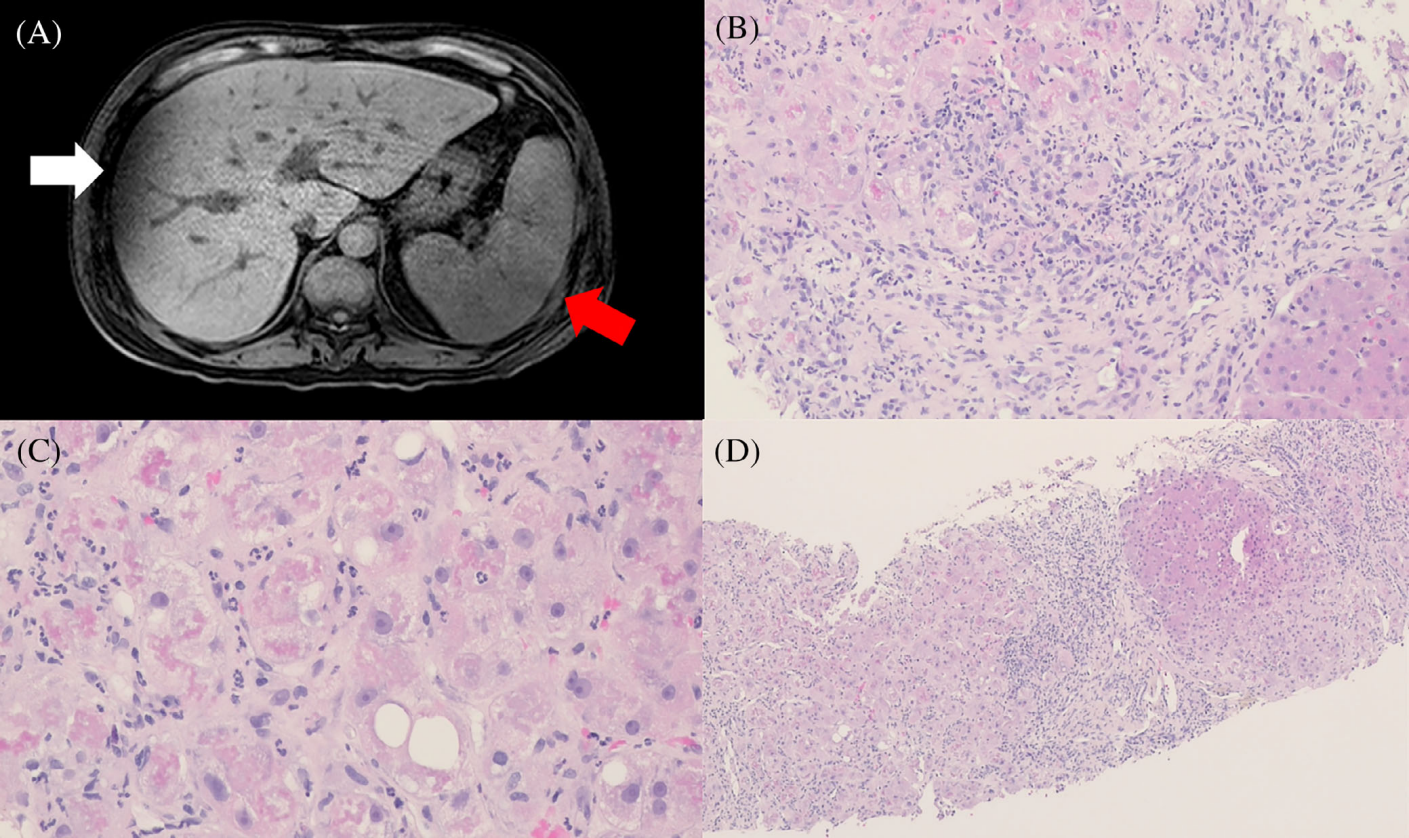
(A) Magnetic resonance cholangiopancreatography shows liver cirrhosis (white arrow) and splenomegaly (red arrow). (B) Microscopic examination shows acute hepatitis with interface activity and neutrophilic infiltration in the fibrotic portal areas and hepatic lobules (HE stain, original magnification 100×). (C) The hepatocytes show ballooning degeneration, mild steatosis, and prominent Mallory bodies (HE stain, original magnification 400×). (D) Marked bridging fibrosis with occasional nodules (Stage 5 fibrosis by Ishak score) (HE stain, original magnification 40×).

*L. monocytogenes* infection predominantly affects the CNS in adults; however, liver involvement is rare. Most reported adult cases occur in those with compromised cell‐mediated immunity^1^ or underlying liver diseases, such as cirrhosis, abscess, hemochromatosis, or chronic active hepatitis.^2^, ^3^, ^4^ Microscopic data on the liver involvement in listeriosis are particularly rare, and the potential mechanisms underlying this association are scarcely addressed in the literature.^5^ Yu et al.^2^ reported three cases of disseminated listeriosis that presented as acute hepatitis, marked by a significant elevation in liver function tests and fever. This case shares similarities with these findings, underscoring the importance of liver biopsy if liver function does not improve despite appropriate antibiotic therapy. Provided the rapid progression to acute liver failure, this case highlights the need for early recognition and intervention in patients with atypical manifestations of *L. monocytogenes* infection.

## CONFLICT OF INTEREST STATEMENT

The authors declare no conflicts of interest.
